# Multiple Exposure and Effects Assessment of Heavy Metals in the Population near Mining Area in South China

**DOI:** 10.1371/journal.pone.0094484

**Published:** 2014-04-11

**Authors:** Ping Zhuang, Huanping Lu, Zhian Li, Bi Zou, Murray B. McBride

**Affiliations:** 1 Key Laboratory of Vegetation Restoration and Management of Degraded Ecosystems, South China Botanical Garden, Chinese Academy of Sciences, Guangzhou, China; 2 Department of Crop and Soil Sciences, Cornell University, Ithaca, New York, United States of America; University of Cincinnati, United States of America

## Abstract

The objective of this study was to investigate the levels of Cd, Pb, Cu and Zn in the environment and several important food sources grown and consumed in the vicinity of Dabaoshan mine in Southern China, and evaluate potential health risks among local residents. The Cd, Pb, Cu and Zn concentrations of arable soils and well water near the mines exceeded the quality standard values. The concentrations of Cd and Pb in some food crops (rice grain, vegetable and soybean) samples were significantly higher than the maximum permissible level. The Cd and Pb concentrations in half of the chicken and fish meat samples were higher than the national standard. The residents living near Dabaoshan mine had higher Cd and Pb levels in hair than those of a non-exposed population. The intake of rice was identified as a major contributor to the estimated daily intake of these metals by the residents. The hazard index values for adults and children were 10.25 and 11.11, respectively, with most of the estimated risks coming from the intake of home-grown rice and vegetables. This study highlights the importance of multiple pathways in studying health risk assessment of heavy metal exposure in China.

## Introduction

Mining and smelting activities have had an important role for local and national economies; however, mining-related industries have commonly been performed in an uncontrolled way, giving rise to severe soil erosion and environmental problems, especially heavy metal pollution [1], [2]. The discharge of acidic mine drainage (AMD), with elevated levels of heavy metals, can contaminate the downstream water, agricultural soils, food crops and biota and pose a health risk to residents near the mining areas [3], [4]. Heavy metal contamination by mining is a major environmental concern on a global scale, particularly in developing countries. Within the global mineral resources industry, China has been one of the largest producers and consumers of several metalliferous and nonmetallic mineral commodities for many years. In China, there are about 8,000 state-owned mining enterprises and 230,000 collectively owned mines that produce hundreds of millions of tons of mining wastes annually [5]. As a result, health related incidents caused by heavy metal pollution in China have risen sharply since 2005, with major accidents attracting nationwide attention [6], [7]. It was reported that high Cd in rice on the Chinese market was mainly the result of contaminated fields affected by AMD. Health risks in mine areas affect not only workers, but the whole population living around the areas, in particular children, so that millions of people in the world are estimated to be exposed to metals in mine areas [8]–[10].

There exist multiple exposure pathways for residents living close to mining or mineral-processing sites, including direct ingestion of soil and water, dermal contact by contaminated soil and water, inhalation of dusts, and consumption of food crops and animals. Various studies have been conducted to evaluate human health risks due to heavy metal exposure through soil [11], water [12], rice [13], [14], vegetables [15], [16], and even dust [17] from metalliferous mining areas throughout the world, such as Romania, Poland, Korea, China, and France. In general, dietary intake has been recognized as the main route of exposure for most populations, although inhalation can play an important role in very contaminated sites [18], [19]. Simple media or pathway-specific approaches to risk assessment may fail to ensure public safety, so it is necessary to apply multi-pathway risk analysis involving all relevant environmental media to identify the dominant pathway of potential concern [9], [20].

For metal toxicity monitoring and human health risk assessment, human hair has been widely used in biomonitoring of heavy metals in recent years to estimate environmental exposure levels and assess nutritional status [21]. As a metabolically inactive tissue, hair has become well established, especially for investigating levels of and changes in many heavy metals that accumulate in the body [22]. Human hair analysis has the advantages over other tissues of being less invasive to sample, more convenient to store and transport, and less hazardous to handle. Furthermore, hair levels of metals are less sensitive to immediate intake and could therefore also be a useful biological indicator in characterizing long-term exposure to the measured metal contaminant.

The aims of this study are: (1) to determine the levels of heavy metal concentrations in the environment and foodstuffs near the mining area, (2) to provide a better understanding of each exposure pathway and evaluate the potential health impacts of these metals on the general population. The results of this assessment will aid development of management options and health intervention policies for the affected areas near metal mining and smelting in China and around the world.

## Materials and Methods

### Ethics Statement

This research was approved by South China Botanical Garden, Chinese Academy of Sciences. The permit for each location was obtained by the authority of Shaxi town. The specific location of field study was provided in the text. The vegetables were purchased from the farmer. All necessary permits were obtained for the described field study. The field studies did not involve endangered or protected species. All participants were informed about the objectives and methods of the study before the investigation. And written consent was obtained from all participants. Data will be made available upon request.

### Study Area

**Figure 1:**
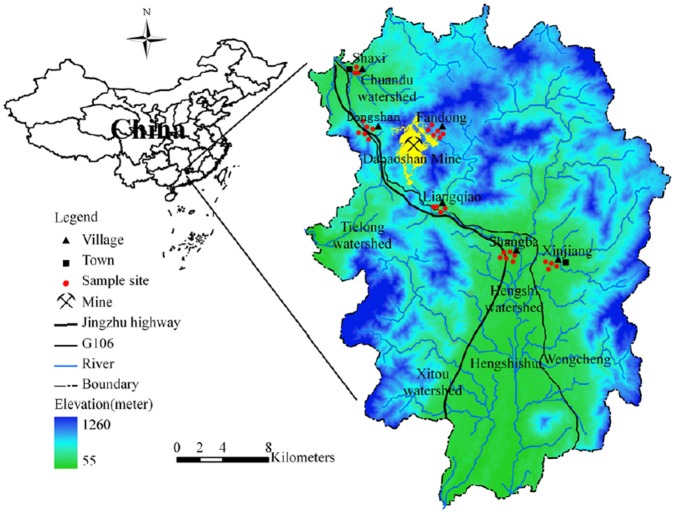
The location of sampling sites in the vicinity of Dabaoshan mine in Guangdong province in South China. The six sampled villages are Shaxi, Dongshan, Fandong, Liangqiao, Shangba and Xinjiang.

Dabaoshan mine (24°31′37″ N; 113°42′49″ E), the largest mine in South China, is located in eastern Shaoguan city, Guangdong, and has been in full-scale operation as a large-scale and integrative quarrying mine since the 1970s (Figure 1). The climate in this area is characterized by a humid subtropical climate, with an annual average temperature of 20.3°C and rainfall of 1762 mm. The Hengshi river (Southward) originates at the mine site and is the main drainage pathway for effluent from the Dabaoshan mine, with the Chuandu river (Northward) being another drainage pathway. The rivers thus deliver significant quantities of heavy metals to numerous villages in this region. After about 40 years of exposure to several heavy metals, some local residents in the mining area have begun to acquire upper gastrointestinal diseases; specifically, oesophageal and stomach cancer are reported to be prevalent. Thus, certain villages around Dabaoshan mine have been termed endemic cancer villages, with a mortality rate approaching 56% in humans [23]. Previous studies in this area have reported that arable soil and foodstuffs in the vicinity of the mine were severely polluted by heavy metals [24] and that there was an increased risk of behavioral problems in school-aged children associated with metal exposures [25]. Wang et al [26] also reported elevated human cancer mortality rates among the metal-exposed populations in Dabaoshan mine area. Six sampling sites near the mine were selected as the study areas (See Supporting Information S1). The agricultural soil in this region was repeatedly irrigated with polluted water from the Hengshi river and Chuandu river. There are about 60–200 households in each village, and they have similar population structures, living conditions and lifestyle.

### Sample Collection

Several different environmental media samples, including 122 soils, 54 rice grains, 320 vegetables, 30 soybeans, 12 sediments of fishponds, 150 fishes, 20 well waters, 48 chickens, and 64 hair samples were collected from different sites around Dabaoshan mine (Table S1). Rice grains and tens of species of home-grown vegetables (Table S2), and their corresponding soil samples were collected from SX, DS, FD, LQ, SB, and XJ villages during November 2007 – May 2008. There are 3–5 replications for each vegetable. Soil samples were collected from the top 0–10 cm layer. Soybean was sampled from DS and FD villages in August 2009. At each site of sampling, three to five subsamples were collected to form a composite sample which was stored in a polyethylene zip-bag, and immediately transported to the laboratory. During April - August 2009, a total of 24 wells were selected and sampled from DS, FD and SB villages because these well waters were consumed by the local residents.

Sediment samples from 3 fishponds at FD village were collected from the surface down to a depth of 10 cm at five different locations, and these samples were pooled together. Six species of fish and sediment samples were sealed in polyethylene bags and kept cold on ice during transportation to the laboratory. The number of fish, total length, fresh weight (fw) and habitat of fish samples from three fishponds are shown in Table S3.

An experimental group of chicken was fed with rice grain (grown in FD contaminated soil), and the control group was fed with chickenfeed (bought from the market). The concentrations of Pb, Cd, Zn and Cu in the metal-enriched rice and chickenfeed fed with chickens are shown in Table S4. After feeding for 6 months, pectoral muscle of all chickens were separately dissected from the body of the specimens, frozen in liquid nitrogen, and freeze-dried. To characterize the exposure level and metal accumulation in human body, hair samples were taken from 24 inhabitants of both DS and FD village (n = 48). Sixteen further hair samples were collected from non-exposed populations. All the study participants gave their permission to be included in the study.

The soil samples were air-dried at room temperature, then pulverized and sieved through a 1 mm stainless-steel mesh. All home-grown food crops were washed thoroughly with Milli-Q water, and the fresh weight of the samples were recorded. All fish and sediment samples were sealed in polyethylene bags and kept cold on ice during transportation to the laboratory. Sediment samples were air-dried, crushed, sieved through a 2 mm screen, then pulverized and passed through a 0.2 mm mesh sieve.

### Analysis of Samples

Four heavy metals (Cd, Pb, Cu and Zn) were analyzed in all the samples. Soil and sediment samples were digested in preparation for total metal analysis using a concentrated acid mixture (HNO_3_, HClO_4_ and HF). For vegetables and soybeans, dried samples were digested with HNO_3_ and HClO_4_ in a 5∶1 ratio until a transparent solution was obtained [27]. Chicken muscle, fish muscle and hair samples were digested in HNO_3_ (16 mol/L) and H_2_O_2_ (30%) by microwave. Each hair sample was cut into smaller sections, thoroughly mixed, and ample portions were washed using the method described by Altshul et al [28]. The concentrations of heavy metals in soil and foodstuff samples were analyzed using an atomic absorption spectrophotometer (AAS, GBC932AA), with the concentrations of Cd and Pb in rice grain, vegetables, fish and chicken being determined using graphite furnace atomic absorption spectrophotometer (GFAAS, GBC932AA). The hair digestion procedure was the same as the method described by Wang et al. [21]. The heavy metal concentrations of hair samples were analyzed with an inductively coupled plasma mass spectrometer (ICP-MS) (Agilent 7700x, Agilent Scientific Technology Ltd., USA).

### Data Calculation

The translocation capability of heavy metals from the soil to the edible part of crops can be described using a bioaccumulation factor (BAF). The BAFs of Cd, Pb, Cu and Zn were calculated as follows:
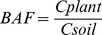
where Cplant and Csoil on dry weight basis represent the heavy metal concentration in edible part of food crops and soils, respectively.

The average estimated daily intake (EDI) of heavy metals by the human subjects was calculated using the following equation, which is recommended by the US EPA [29].
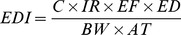
where EDI is the average daily intake or dose through ingestion (μg/kg bw/day); C is the heavy metal concentration in the exposure medium (mg/L or mg/kg); IR is the ingestion rate (L/day, or kg/day); EF is the exposure frequency (365 days/year); ED is the exposure duration (70 years, equivalent to the average lifespan); BW is the body weight (kg). Average adult and child body weights were considered to be 60 and 30 kg, respectively and AT is the time period over which the dose is averaged (365 days/year times number of exposure years, assumed to be 70 years in this study). A questionnaire-based survey was conducted in the studied villages to determine key risk factors such as dietary behaviors, daily activities and lifestyle of local people. We invited 50 local residents in each village to participate in the survey. In this study area, most foodstuffs are self-produced, whereas pork is purchased from the market. The average considered daily intakes of adults and children is shown in Table S5, according to the survey and reports by Ma et al. [30] and Zhai et al. [31].

The human health risk posed by heavy metal exposure are usually characterized by the target hazard quotient (THQ) [29], the ratio of the average estimated daily intake resulting from exposure to site media compared to the reference dose (RfD) for an individual pathway and chemical. Oral reference dose obtained from the Integrated Risk Information System [32], is an estimation of maximum permissible risk to a human population through daily exposure when taking into consideration a sensitive group during a lifetime. The applied RfD for Cd, Pb, Cu and Zn was 1.0, 4.0, 40, 300 μg/kg/d, respectively. The THQ based on non-cancer toxic risk is determined by
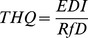
If the value of THQ is less than 1, the risk of non-carcinogenic toxic effects is assumed to be low. When it exceeds 1, there may be concerns for potential health risks associated with overexposure.

To assess the overall potential risk of adverse health effects posed by more than one metal, the THQs can be summed across contaminants to generate a hazard index (HI) to estimate the risk of a mixture of contaminants. The HI refers to the sum of more than one THQ for multiple substances and/or multiple exposure pathways. In the present study, the HI was used as a screening value to identify whether there is significant risk caused by heavy metals through average dietary consumption for the residents living near the Dabaoshan mine.

### Quality Control

Appropriate quality assurance procedures and precautions were carried out to ensure reliability of the results for the different environmental media under investigation. Double distilled deionised water was used throughout the study. Glassware was properly cleaned, and the reagents were of analytical grade. Reagent blank determinations were used to correct the instrument readings. For validation of analytical procedures, a recovery study was carried out by spiking and homogenizing several already analyzed samples with varying amounts of standard solutions of the metals. Several standard reference materials (SRM) were obtained from the National Research Center for CRMs (Table S6) and used for validation of the analytical procedure. Blank and drift standards were run after every twenty determinations to maintain instrument calibration. The coefficient of variation of replicate analyses was determined for the measurements to calculate analytical precision.

## Results and Discussion

### Heavy Metals in Different Environmental Media and Food Chain

**Figure 2:**
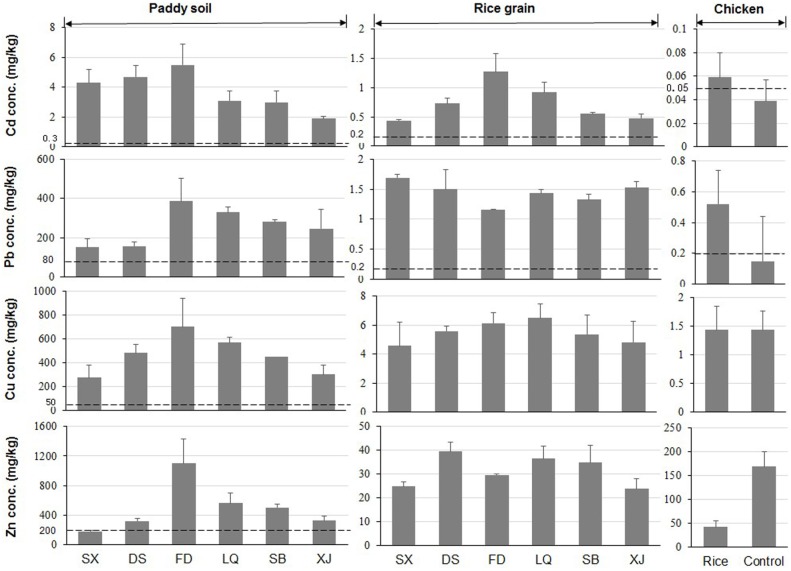
Comparison of concentrations (mg/kg, mean ± SD) of Cd, Pb, Cu and Zn and their respective Chinese national quality value (dotted line) in paddy soil, rice grain and chicken muscle samples collected at different localities in the vicinity of Dabaoshan mine. The number of samples is 9 for respective paddy soil, 8–10 for rice grain, 24 for each group chicken. For soil and rice samples, they were collected from six specific locations, including Shixi (SX), Dongshan (DS), Fandong (FD), Liangqiao (LQ), Shangba (SB) and Xinjiang (XJ). The chicken was fed with rice grain collected from Fandong village.

**Figure 3:**
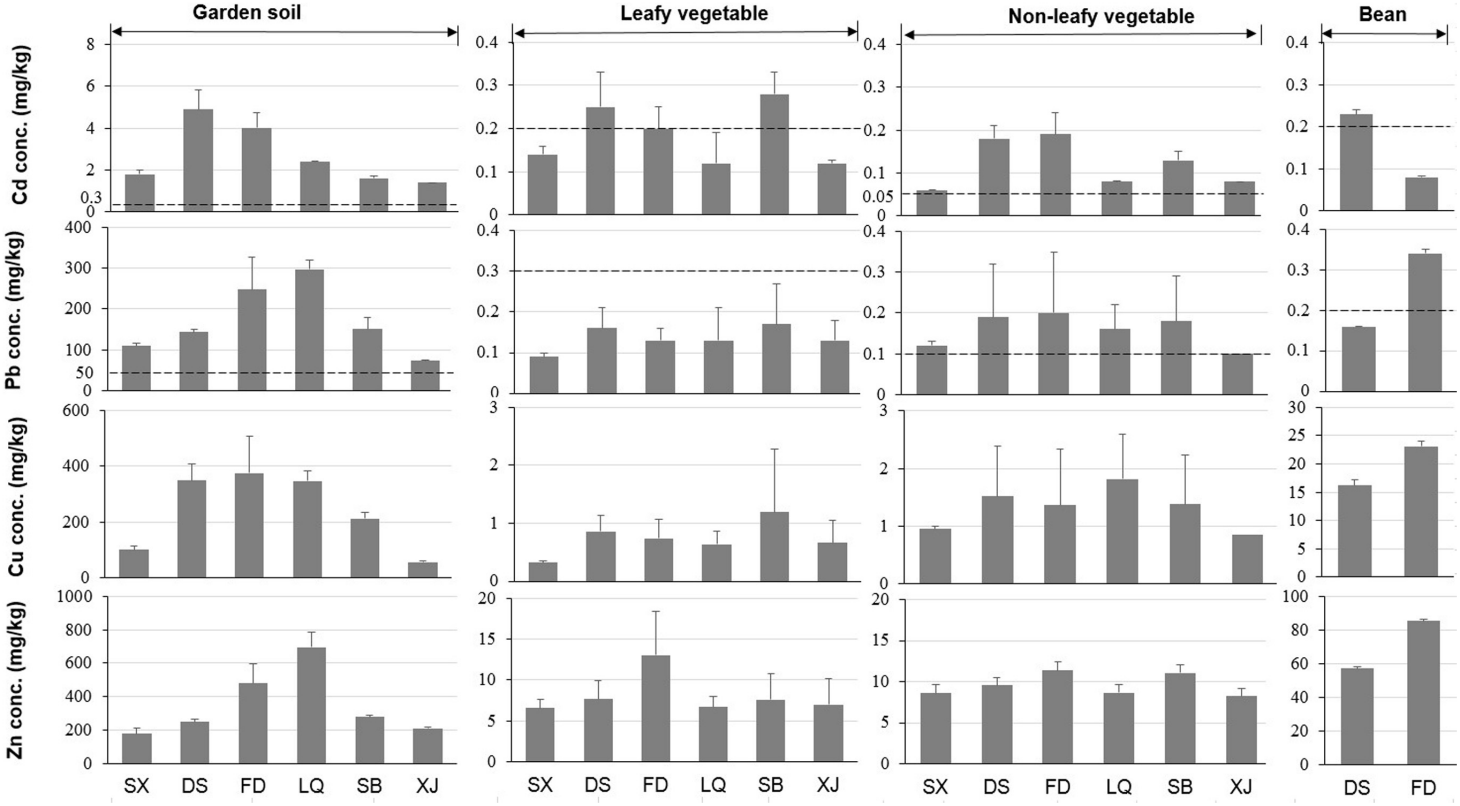
Comparison of concentrations (mg/kg, mean ± SD) of Cd, Pb, Cu and Zn and their respective Chinese national quality value (dotted line) in garden soil, leafy vegetables, non-leafy vegetables and bean samples from different locations. The number of samples is 9 for respective garden soil, 3–5 for each vegetable, 15 for respective soybean. For soil and vegetable samples, they were collected from six specific locations, including Shixi (SX), Dongshan (DS), Fandong (FD), Liangqiao (LQ), Shangba (SB) and Xinjiang (XJ). The bean samples were collected from DS and FD villages.

The environmental sample analysis results showed widespread heavy metal (Cd, Pb, Cu and Zn) contamination in the different exposure media around the Dabaoshan mine (Figures 2–3). Among the six sites in the study area, the paddy soil samples collected from FD showed the highest concentrations of Cd (5.5 mg/kg), Pb (386 mg/kg), Cu (703 mg/kg), and Zn (1100 mg/kg), presumably because FD is located on the mountaintop of the Dabaoshan mine (Figure 2). In contrast, the soil samples collected from XJ, which is located far from Dabaoshan mines (>15 km), showed the lowest heavy metal concentrations. These results suggest that elevated heavy metal concentrations in soils are associated with the mining activities, and indicate that this area is unsuitable for agricultural use. The highest Cd concentration (4.9 mg/kg) in garden soils was found in DS village, whereas soil Pb (297 mg/kg) from LQ was significantly higher compared with the other sites. Concerning co-located arable soils vegetated by various food crops, generally the paddy soil contained higher heavy metal concentrations than the garden soil. This may be due to the fact that the paddy soils were irrigated with highly heavy metal-contaminated stream water whereas the water sources of the garden soils were mainly derived from well water or rainfall.

In comparison to Chinese soil quality guideline values, the soil concentrations of Cd, Pb, Cu and Zn were frequently exceeded (Figures 2–3). Distance from the mine was a key determinant of soil pollution, as there are significant spatial differences among these six studied villages. This finding was consistent with the previous studies [3]. The heavy metal concentrations in soil samples around Dabaoshan mine were remarkably high in general, being comparable with those recorded near a Pb–Zn mine of Spain [33], and higher than those reported for the Songcheon Au–Ag mine in Korea [34], an old mining area in Romania [15] and an abandoned mine in Thailand [35]. From Figures 2–3, it is clear that the characteristics of soil significantly affect the soil level at which the heavy metals will exceed recommended limits in food crops and pose risks in terms of food safety and animal health.

The heavy metal concentrations in rice grain from the six sites decreased in the order Zn>Cu>Pb>Cd (Figure 2). The maximum concentrations of Cd (1.27 mg/kg) at FD and Pb (1.69 mg/kg) at SX were approximately 6–8 folds greater than the maximum permissible level for both metals of 0.2 mg/kg of rice [36]. The relatively high heavy metal concentrations measured in the self-produced food crops were generally consistent with the elevated heavy metal concentrations found in the paddy and garden soils, although the consistently elevated Pb in rice grain did not follow the site-to-site variation of soil Pb concentration (Figure 2). In comparison with other mining areas, the highest Cd concentration (1.27 mg/kg) in rice grain collected from the present study area was 6–8 times higher than concentrations reported by Lee (0.15 mg/kg, in the Au-Ag-Pb-Zn mining area) [37] and by Ji (0.224 mg/kg, near abandoned metal mine) [38] from Korea, and about 5 times lower than those recorded in the Chenzhou Pb/Zn mining area from China [13], i.e. 6.99 mg/kg. The Pb concentrations in rice grain were higher than those reported for a Pb/Zn mining area from China (0.8 mg/kg) [13] and those reported for an abandoned metal mine from Korea [38].

The concentrations of heavy metals in garden soils and self-produced vegetables (e.g., mustard, Chinese cabbage, lettuce, spinach, garden pea, and tuber of sweet potato, etc. see Table S2) are presented in Figure 3. The leafy and non-leafy vegetables contained higher heavy metal concentrations, especially of Cd, in villages DS, FD and SB. Comparison of the results with the food quality guidelines as set by the Chinese government indicated that the Cd limit was exceeded in 60% of the vegetable samples. For soybean, the Cd concentration in DS and Pb concentration in FD (Figure 3) exceeded the maximum permissible level of 0.2 mg/kg [36]. The concentrations of heavy metals in homegrown vegetable samples around Dabaoshan mine were comparable with those reported in an old mining area of Romania [15], and higher than what was recorded in an abandoned metal mine area of Korea [38]. Of all the vegetables tested, leafy vegetables always contained higher Cd than the non-leafy vegetables (Figure 3).

Contamination of local wetlands, ponds, and rivers by sluicing waste and AMD could provide a pathway for heavy metals into the aquatic food chain [39]. The Hengshi and Chuandu Rivers are the source of drinking and irrigation water for the residents near Dabaoshan mine, and groundwater also may be contaminated by the two rivers. The concentrations of heavy metals in the well water collected from DS, FD and SB villages exceeded the national standard (Table 1), indicating the drinking water in the local private wells were contaminated by acidic mine water from the Dabaoshan mine, which agreed with previous results [40]. In view of these results, a public water supply system was installed and the community was advised not to use private wells.

**Table 1:** Heavy metal concentrations (mg/L, mean ± SD) in well water sampled from three sites near Dabaoshan mine.

	SB	DS	FD	National standard^a^
Cd	0.0095±0.000	0.01±0.001	0.015±0.001	0.005
Pb	0.013±0.001	0.008±0.000	0.017±0.008	0.01
Cu	1.42±0.012	1.29±0.031	1.58±0.091	1.0
Zn	3.63±0.82	3.47±0.18	4.81±1.05	1.0

a Standards for drinking water quality (GB5749-2006) set by the Ministry of Health of the People’s Republic of China.

Heavy metal analyses in sediments taken from three different sites at FD village show that the levels of Cd (0.32–8.91 mg/kg), Pb (262–327 mg/kg), Cu (239–1477 mg/kg) and Zn (386–4524 mg/kg) were markedly higher than normal background levels (Figure S1). The heavy metal levels of fodder (Ryegrass) cultivated around the fishponds and fed to fish were 1.65, 3.95, 18.02 and 88.01 mg/kg fw for Cd, Pb, Cu and Zn, respectively. It is a positive result that average Cd, Cu and Zn concentrations in fish muscle tissue were within maximum permissible levels. However, the concentrations of Pb in more than 60% of the fish fresh samples from site 1 were above the maximum permissible level (Figure S1). These results suggested the high translocation of metals in site 1 were likely due to the low sediment pH values (5.22), which might cause the heavy metals to be more soluble and bioavailable [41]. Elevated concentrations of heavy metals in the water environment and its human health risk as a consequence of historical mining have been reported elsewhere all over the world [42], [43]. Therefore, mining effluents increase metal levels in the aquatic system, and consumption by fish of contaminated sediments and water originating from mining operations is an important exposure route that can result in metal accumulation in fish.

**Figure 4:**
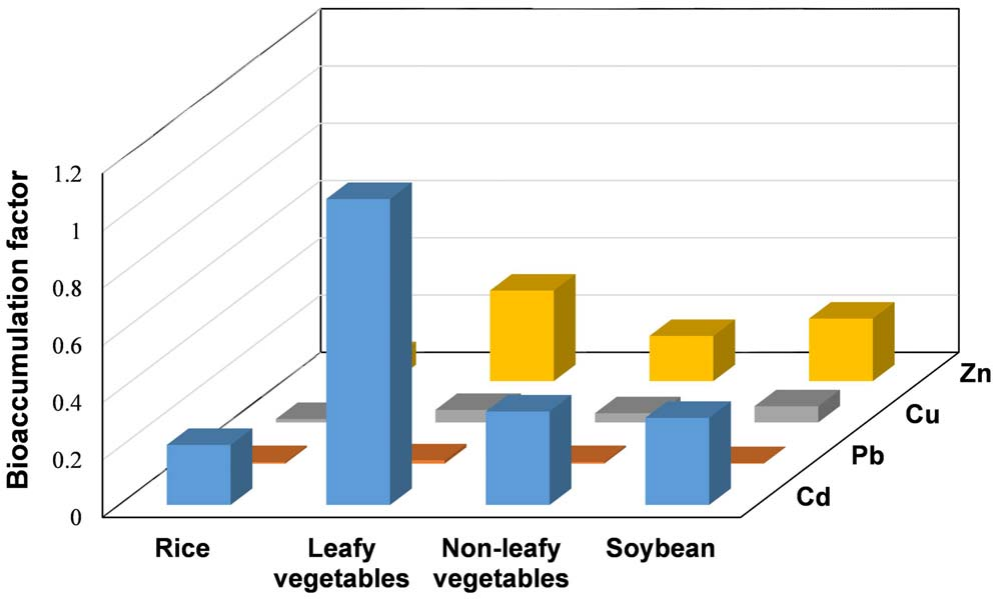
Bioaccumulation factor (BAF), a ratio of heavy metals concentration in the edible part of rice, leafy vegetables, non-leafy vegetables and soybean to that in the corresponding soil.

Soil-to-plant transfer is one of the key components of human exposure to metals through the food chain. As shown in Figure 4, large variations in BAFs were observed among different food crops and metals. The results showed that BAFs of all food crops for these tested metals were in the order: Cd>Zn>Cu>Pb, consistent with a report by Li et al. [44]. The BAF values for Cd varied from 0.21 to 1.07, with the highest level in leafy vegetables. Leafy vegetables usually grow quickly and have high transpiration rate, which was in agreement with previous study [45]. The high BAFs of Cd and Zn for respective environmental media were similar to the results for some crops from Chenzhou Pb/Zn mine reported by Liu et al. [13]. Generally, the BAF of heavy metals is controlled by the chemical speciation of heavy metals in soil, soil properties, such as pH and salinity, and plant physiological features [46], [47]. In the present study, the lower pH in the sandy soil (Table S7) can increase the solubility of heavy metal and may transfer into the crop tissues.

In the third food chain exposure route investigated (soil-rice-chicken), it is notable that Cd and Pb concentrations in chicken muscle fed with metal-contaminated rice grain (grown in contaminated soil at FD) were higher than those of the control (Figure 2). The levels of Cd and Pb in muscle of chicken fed with contaminated rice exceeded the maximum permissible levels for Cd and Pb in meat prescribed by China. For livestock, the ingestion of rice or grass is the most relevant pathway for the intake of heavy metals [48]. From the data produced in the present study, it is clear that cultivation of food crops on contaminated soil for human or livestock consumption can potentially lead to the uptake and accumulation of trace metals in the edible plant parts, with a resulting risk to human and animal health in the Dabaoshan mining area.

### Exposure Assessment

Human exposure to heavy metals in soils near the Dabaoshan mine might occur directly through the ingestion of soil or indirectly through consumption of locally grown vegetables and dairy and meat products from locally raised farm animals. Table 2 summarizes the results of the actual exposure assessment for adults and children exposed to heavy metals through five exposure pathways around Dabaoshan mine. For adults, the total EDI were 5.45, 10.59, 45.14 and 278.38 μg/kg bw/d for Cd, Pb, Cu and Zn, respectively, which were much higher than those reported for an abandoned metal mine from Korea [38]. The EDIs of Cd, Pb and Cu for adults through these five exposure pathways were 5.45, 2.64 and 1.13 fold higher than the RfD of these metals, respectively. It should be cautioned that the EDIs of heavy metals for children were slightly higher than those for adults. The biggest contribution to the intake of heavy metals came from rice, with tens of times as much as intake through others ingestion pathway, as shown in Table 2.

**Table 2:** Estimated daily intake (EDI, ug/kg bw/d) and target hazard quotient (THQ) for adult and children exposed to contaminants in the vicinity of Dabaoshan mine.

Metal	Pathways	EDI		THQ	
		Adult	Child	Adult	Child
Cd	Ingestion of rice	4.54	4.83	4.54	4.83
	Ingestion of vegetables				
	Leafy vegetables	0.54	0.42	0.54	0.42
	Non-leafy vegetables	0.26	0.21	0.26	0.21
	Bean	0.04	0.08	0.04	0.08
	Ingestion of meat			0.03	0.04
	Fish	0.03	0.04		
	Chicken	0.02	0.02	0.02	0.02
	Ingestion of soil	0.00	0.02	0.00	0.02
	Ingestion of water	0.02	0.02	0.04	0.04
	Total	5.45	5.63	5.47	5.65
Pb	Ingestion of rice	8.93	9.50	2.23	2.38
	Ingestion of vegetables				
	Leafy vegetables	0.39	0.31	0.10	0.08
	Non-leafy vegetables	0.24	0.19	0.06	0.05
	Bean	0.07	0.13	0.02	0.03
	Ingestion of meat				
	Fish	0.56	0.57	0.14	0.14
	Chicken	0.16	0.21	0.04	0.05
	Ingestion of soil	0.22	1.30	0.05	0.32
	Ingestion of water	0.02	0.02	0.01	0.00
	Total	10.59	12.23	2.65	3.06
Cu	Ingestion of rice	34.11	36.31	0.85	0.91
	Ingestion of vegetables				
	Leafy vegetables	2.13	1.67	0.05	0.04
	Non-leafy vegetables	1.67	1.34	0.04	0.03
	Bean	5.57	10.48	0.14	0.26
	Ingestion of meat				
	Fish	0.85	0.86	0.02	0.02
	Chicken	0.43	0.57	0.01	0.01
	Ingestion of soil	0.39	2.32	0.01	0.06
	Ingestion of water	2.69	2.15	0.07	0.05
	Total	45.14	53.55	1.20	1.39
Zn	Ingestion of rice	194.86	207.43	0.65	0.69
	Ingestion of vegetables				
	Leafy vegetables	23.45	18.33	0.08	0.06
	Non-leafy vegetables	14.62	11.70	0.05	0.04
	Bean	20.23	38.08	0.07	0.13
	Ingestion of meat				
	Fish	4.74	3.39	0.02	0.01
	Chicken	12.60	16.80	0.04	0.06
	Ingestion of soil	0.41	2.49	0.00	0.01
	Ingestion of water	7.46	5.96	0.02	0.02
	Total	278.38	304.17	0.93	1.02
Hazard index				10.25	11.11

Health risks to residents in the study area through the consumption of agricultural products, and through inadvertent ingestion of soil were assessed by estimating target hazard quotients (THQ). A THQ value greater than 1 would indicate that a potential health risk may exist. The THQs of heavy metals from multiple consumption pathways is in decreasing order Cd>Pb>Cu>Zn (Table 2). The THQs of all heavy metal in rice were the highest among all the crops, which higher than those grown on reclaimed tidal flat soil in the Pearl River Estuary [44]. Considering that rice is a staple food in the diet of the people in the area, intake of heavy metals through rice consumption is likely to be a main source of heavy metal intake among residents in the area. The HI values (the sum of all THQs) through diet and soil for adults and children in the Dabaoshan mine area were 10.25 and 11.11, respectively; both are higher than reported in other areas [16], [49]. These results suggest that the adults and children living around the Dabaoshan mine may experience adverse health effects. Considering THQ determination for the different metals and pathways for children, besides rice, the highest risk was for ingestion of vegetables for Cd (0.71), followed in descending order by ingestion of soil for Pb (0.32) and ingestion of bean for Cu (0.26). Concern has focused particularly on children who become exposed to heavy metals to a greater extent than adults, which may harm brain and nervous system development [50]. Despite Zn having the highest concentration among the metals, its THQ value was below 1, indicating that Zn does not pose a health risk to the local residents.

Mining sites are usually characterized by soil contamination and a higher exposure to heavy metals for the population residing in the area [51], [52]. Exposure level and risk assessment in the study area are compared with those of other studies [9], [15], [35], [38], [49], [53]–[56] from mining and smelting sites around the world as shown in Table 3. The health risk for residents living in the vicinity of Dabaoshan mine associated with the consumption of local foodstuffs was markedly higher than that reported for other metal mines all over the world. Some evidences also revealed a large part of the population in the vicinity of Dabaoshan mine had contacted or ingested contaminated soil [24], [57], contaminated water [23], [40], consumed local agricultural products, such as foodcrops [24], fish [58] and chicken, over a long period of time. These concerns were further supported by the finding of behavioral problems for school-aged children [25] and significantly elevated blood levels of metals (38.9 and 24.1 μg/L for Pb and Cd in high exposed area vs 4.46 and 1.87 μg/L for Pb and Cd in low exposed area) [26] in local residents from the same mining area. Liu et al. [23] and Wang et al. [26] reported that an increased risk of mortality from all cancer (e.g. enteron tumors) was probably associated with long-term environmental exposure to both Cd and Pb.

**Table 3:** Comparison of estimated dietary intake (EDI) and hazard index (HI) of heavy metals in different areas around the world.

Sites	EDI (ug/kg bw/d)or HI	Cd	Pb	Cu	Zn	References
Dabaoshan mine, Shaoguan, China	EDI	5.45–5.63	10.6–12.2	50.4–59.1	292–320	In this study
	HI	5.45–5.63	2.65–3.06	1.26–1.48	0.97–1.07	
Abandoned metal mine, Kanchanaburi, Thailand	EDI	0.25–5.34	0.71–1.46			[35]
	HI	0.25–5.34	0.18–0.36			
Abandoned metal mine, Katowice, Poland	HI	2.6–2.7		0.025–0.041	0.11–0.18	[56]
Old mining area, Banat, Romania	HI	<1	>1	<1	<1	[15]
Lead-zinc mining area, Jiangsu, China	HI	1.81–3.32	3.01–16.2	0.164–0.189	0.28–0.30	[9]
Huludao Zn plant, Liaoning, China	EDI	0.70	1.36	45.5	204	[49]
	HI	0.749	0.364	1.22	0.731	
Pb/Sb smelter, Nanning, China	HI	7.42	1.35	0.146	0.323	[53]
Chatian mercury mining deposit Hunan, China	EDI	0.05–1.66	0.359–1.6			[55]
	HI	0.05–1.66	2.67–2.99			
Songcheon Au-Ag mine, Gangneung-si, Korea	HI	0.75		0.078	0.26	[54]
Abandoned metal mine, Korea	EDI	0–0.195	0.001–0.233	0.009–13.4	0.208–66.6	[38]
	HI	0.179	0.077	0.027	0.063	

### Source Allocation for Different Metals

**Figure 5:**
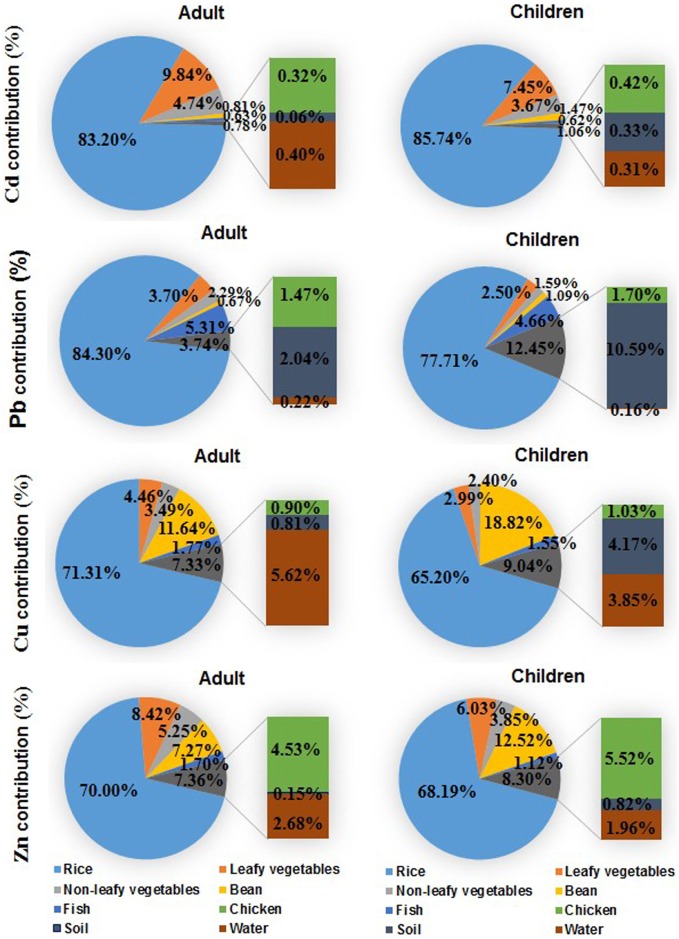
Relative contribution profile of daily human intake. Relative contribution was derived by dividing the daily intake estimate from a given source by the total daily intake estimate from all of the sources under investigation.

When the profiles of relative contribution of soil and foodstuffs to daily heavy metal intake estimates from all the sources being investigated (e.g., rice grain, vegetables, beans, fish, chicken, water and soil) were compared between adults and children (Figure 5), intake of rice was the major source of Cd, Pb, Cu and Zn exposure, accounting for 65.2–85.7% of the estimated total daily intake in the study area. Interestingly, soil ingestion was a second important factor for daily Pb intake, accounting for 10.6% of children’s exposure. Among all the main exposure pathways, the most important exposure pathway for heavy metals appeared to be the ingestion of locally grown rice, with about several times as much as intake through vegetables. The allocations of Cd and Pb from dietary exposure were comparable with that reported for Pb by Dong and Hu [20], but higher than the contribution from diet of 70% and 50% for Cd and Pb, respectively, estimated by Plant et al. [18].

The results of this study suggested that the contribution to total Cd and Pb exposure from drinking water is even lower than 1%, which was consistent with the finding by Dong and Hu [20]. It is clear that the allocation of dietary exposure is the highest in the study area, and the contributions by the soil and water exposure pathways are relatively low. Specifically for children, a health risk from Pb was indicated, with the second most important exposure pathway being the direct ingestion of soil. The source allocation of Pb for soil accounted for 10.6% of children’s exposure, which was similar to that reported for an abandoned metal mine area of Korea (11.1–16.8%) [38]. Staessen et al. [19] also found that 2–4% of the variance in the long-term body burden of Cd was directly related to consumption of vegetables, implying that the ingestion of locally grown vegetables is an important source for human Cd exposure.

Compared to other countries, the relatively high source contribution from the diet in China is due to the high concentrations of heavy metals in the food. The high heavy metal concentrations in grains and vegetables were mainly due to enrichment from the soil [59]. The substantial contribution of rice and soil to the intake of four heavy metals indicate that the status of metal contamination of soil and rice should receive further attention in the metal mine areas throughout the country. In fact, the potential health risk from heavy metals can be for some individuals much higher than our calculations based on average ingestion of water, food and soil. Some local inhabitants, who consume more contaminated locally grown food crops, breathe contaminated air, or smoke, might be exposed to a health risk from dietary heavy metals well above the calculated risk. As a result, great effort is required to control concentrations of heavy metals in soil to reduce dietary metal exposure, and ultimately to eliminate heavy metal exposure in China.

### Heavy Metal Levels in Hair and Global Health Implications

**Figure 6:**
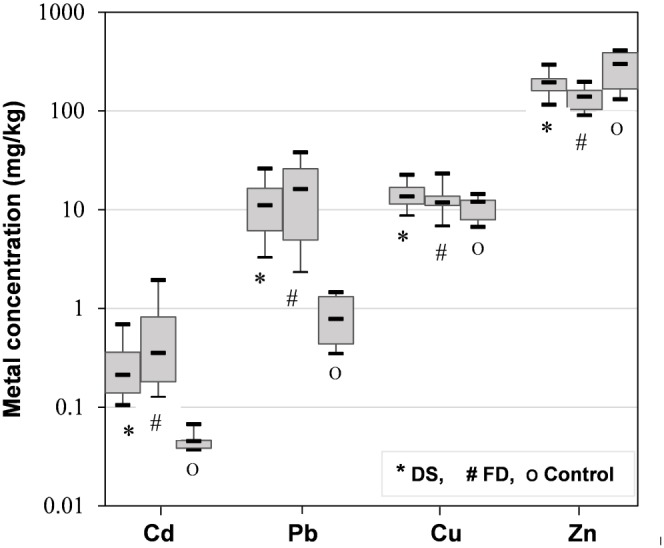
Box-plot diagram of four elements in hair samples from two villages (Dongshan, DS, Fandong, FD) in the vicinity of Dabaoshan mine and the control area (non-exposed site). Y-axis is presented in logarithmic scale. The central mark on each box is the median with the edges of the 25th and 75th percentiles.

Human hair can serve as a useful direct biomonitoring tool to assess the extent of heavy metal exposure to residents in metal-polluted areas. The heavy metal concentrations in the hair samples are shown in Figure 6. The hair levels of four heavy metals were in the order of Zn>Pb, Cu>Cd. The hair samples from the residents living in the Dabaoshan mine area (DS and FD villages) contained markedly higher average Cd, Pb and Cu concentrations than those of non-exposed populations, with Cd around 5–11 times higher (0.27–0.57 mg/kg) and Pb 15–28 times higher (12.5–23.9 mg/kg) than the non-exposed levels, which might be a consequence of long term exposure of the local residents to the mining activities. Some subjects in FD showed even higher levels of Cd and Pb, with the maxima estimated at 1.94 and 38 mg/kg, respectively. The average levels of hair Pb, Cd, Cu and Zn in the study area were lower than the levels in the scalp hair of adults from S. Domingos mine, Portugal [60], but higher than those recorded in a lead-zinc mining area from China [9]. Interestingly, the Zn concentration in hair of a non-exposed population was higher than that of subjects living near the Dabaoshan mine, which might be due to the higher concentration of toxic metals like Cd and Pb in the vicinity, which can interact with Zn and replace it in the heme enzymes and metallothioneins [61]. Consistent with the higher THQ value of the residents in FD village, their hair samples exhibited higher than those in DS village. This finding agreed well to the conclusions of previous studies [9]. Therefore, the elevated hair Cd and Pb levels provide an independent indicator that residents in the Dabaoshan mine region might be at high risk of toxic metal exposure due to the elevated levels of heavy metals in food crops, fish or livestock.

Increasing evidence shows heavy metal pollution of mined areas to cause health damage to the local inhabitants [4], [26]. For example, itai-itai disease was caused by Cd poisoning in Japan due to the Kamioka mine releasing this metal into river water that was then used to irrigate rice paddy soils [62]. Moreover, tens of thousands of Kabwe’s residents (Zambia) suffered from severe lead poisoning, which resulted from artisanal re-mining of and exposures to wastes from historical lead-zinc mining and smelting [63]. In northern Nigeria, confirmed deaths of at least 400 children were the result of artisanal processing of lead-rich gold ores [64]. There is some evidence to suggest that local inhabitants may develop a natural tolerance to the high soil metal concentrations encountered in their environment, since non-local people who have supposedly suffered from heavy metal poisoning caused by the consumption of home produced vegetables grown in gardens reclaimed from former mine dumps [61], [65]. Thus, by understanding human exposure pathways for toxic metals, the routes by which heavy metals may enter the body and cause health effects, scientists can help identify other mining areas that may pose the highest risk for heavy metal poisoning and need for medical surveillance and intervention.

## Supporting Information

Figure S1 **Comparison of concentrations (mg/kg) of Cd, Pb, Cu and Zn and their respective Chinese national quality value (dotted line) in muscle samples from three different fishponds at Fandong village.** Y-axis of Pb, Cu and Zn concentrations are presented in logarithmic scale.
(EPS)

Table S1 **Number of soil, rice, vegetable, soybean, water, sediment, fish and chicken samples collected from different sampling sites in the present study.**
(DOCX)

Table S2 **The edible part and water content of vegetables.**
(DOCX)

Table S3 **The number of fish, total length, fresh weight (fw) and habitat of fish samples.**
(DOCX)

Table S4 **Heavy metal concentrations (mg/kg, mean** ± **SD) in the metal-enriched rice grain and chickenfeed for both groups of chicken.**
(DOCX)

Table S5 **Average considered daily intakes of adults and children in the present study.**
(DOCX)

Table S6 **Standard reference materials for respective sample under investigation.**
(DOCX)

Table S7 **The pH and organic matter (OM) of paddy and garden soils from Dabaoshan mine.**
(DOCX)

Supporting Information S1 **Description of study area.**
(DOCX)

## References

[1] Rybicka EH (1996) Impact of mining and metallurgical industries on the environment in Poland. Appl Geochem 11: 3–9.

[2] Dudka S, Adriano DC (1997) Environmental impacts of metal ore mining and processing: a review. J Environ Qual 26: 590–602.

[3] Benin AL, Sargent JD, Kalton M, Roda S (1999) High concentrations of heavy metals in neighborhoods near ore smelters in northern Mexico. Environ Health Perspect 107: 279–184. 10.1289/ehp.99107279 PMC1566526 10090706

[4] Plumlee GS, Morman SA (2011) Mine wastes and human health. Element 7: 399–404.

[5] Shu WS, Zhang ZQ, Lan CY (2000) Strategies for restoration of mining wastelands in China. Ecol Sci 19: 24–29.

[6] Gao Y, Xia J (2011) Chromium contamination accident in China: viewing environment policy of China. Environ Sci Technol 45: 8065–8056. 10.1021/es203101f 21928806

[7] Zhang XW, Yang LS, Li YH, Li HR, Wang WY, et al (2012) Impacts of lead-zinc mining and smelting on the environment and human health in China. Environ Monit Assess 184: 2261–2273. 21573711 10.1007/s10661-011-2115-6

[8] Plumlee GS, Durant JT, Morman SA, Neri A, Wolf RE, et al (2013) Linking geological and health sciences to assess childhood lead poisoning from Artisanal gold mining in Nigeria. Environ Health Perspect 121: 744–750. 23524139 10.1289/ehp.1206051 PMC3672918

[9] Qu CS, Ma ZW, Yang J, Liu Y, Bi J, et al (2012) Human exposure pathways of heavy metals in a Lead-Zinc mining area, Jiangsu Province, China. PLoS ONE 7: e46793. 23152752 10.1371/journal.pone.0046793 PMC3496726

[10] Wcisło E, Ioven D, Kucharski R, Szdzuj J (2002) Human health risk assessment case study: an abandoned metal smelter site in Poland. Chemosphere 47: 507–515. 11996126 10.1016/s0045-6535(01)00301-0

[11] Kapusta P, Szarek-Łukaszewska G, Stefanowicz AM (2011) Direct and indirect effects of metal contamination on soil biota in a Zn-Pb post-mining and smelting area (S Poland). Environ Pollut 159: 1516–1522. 21477907 10.1016/j.envpol.2011.03.015

[12] Park JH, Choi KK (2013) Risk assessment of soil, water and crops in abandoned Geumryeong mine in South Korea. J Geochem Explor 128: 117–123.

[13] Liu HY, Probst A, Liao BH (2005) Metal contamination of soils and crops affected by the Chenzhou lead/zinc mine spill (Hunan, China). Sci Total Environ 339: 153–166. 15740766 10.1016/j.scitotenv.2004.07.030

[14] Zhu YG, Sun GX, Lei M, Teng M, Liu YX, et al (2008) High percentage inorganic arsenic content of mining impacted and nonimpacted Chinese rice. Environ Sci Technol 42: 5008–5013. 18678041 10.1021/es8001103

[15] Harmanescu M, Alda LM, Bordean DM, Gogoasa I, Gergen I (2011) Heavy metals health risk assessment for population via consumption of vegetables grown in old mining area; a case study: Banat County, Romania. Chem Cent J 5: 64. 22017878 10.1186/1752-153X-5-64 PMC3212802

[16] Wang X, Sato T, Xing B, Tao S (2005) Health risk of heavy metals to the general public in Tianjin, China via consumption of vegetables and fish. Sci Total Environ 350: 28–37. 16227070 10.1016/j.scitotenv.2004.09.044

[17] Kerin EJ, Lin HK (2010) Fugitive dust and human exposure to heavy metals around the Red Dog Mine. Rev Environ Contam Toxicol 206: 49–63. 20652668 10.1007/978-1-4419-6260-7_3

[18] Plant J, Smith D, Smith B, Williams L (2001) Environmental geochemistry at the global scale. Appl Geochem 16: 1291–1308.

[19] Staessen JA, Vyncke G, Lauwerys RR, Roels HA, Celis HG, et al (1992) Transfer of cadmium from a sandy acidic soil to man: a population study. Environ Res 58: 24–34. 10.1016/s0013-9351(05)80202-6 1350763

[20] Dong ZM, Hu JY (2012) Development of lead source-specific exposure standards based on aggregate exposure assessment: Bayesian inversion from biomonitoring information to multipathway exposure. Environ Sci Technol 46: 1144–1152. 22142206 10.1021/es202800z

[21] Wang T, Fu JJ, Wang YW, Liao CY, Tao YQ, et al (2009) Use of scalp hair as indicator of human exposure to heavy metals in an electronic waste recycling area. Environ Pollut 157: 2445–2451. 19346038 10.1016/j.envpol.2009.03.010

[22] Druyan ME, Bass D, Puchyr R (1998) Determination of reference ranges for elements in human scalp hair. Biol Trace Elem Res 62: 183–187. 9676882 10.1007/BF02783970

[23] Liu YS, Gao Y, Wang KW, Mai XH, Chen GD, et al (2005) Etiologic study on alimentary tract malignant tumor in villages of high occurrence. China Trop med 5: 1139–1141 (in Chinese)..

[24] Zhuang P, McBride MB, Xia HP, Li NY, Li ZA (2009) Health risk from heavy metals via consumption of food crops in the vicinity of Dabaoshan mine, South China. Sci Total Environ 407: 1551–1561. 19068266 10.1016/j.scitotenv.2008.10.061

[25] Bao QS, Lu CY, Song H, Wang M, Ling WH, et al (2009) Behavioural development of school-aged children who live around a multi-metal sulphide mine in Guangdong province, China: a cross-sectional study. BMC Publ Health 9: 217. 10.1186/1471-2458-9-217 PMC2717083 19573251

[26] Wang M, Song H, Chen WQ, Lu CY, Hu QS, et al (2011) Cancer mortality in a Chinese population surrounding a multi-metal sulphide mine in Guangdong province: an ecologic study. BMC Publ Health 11: 319. 10.1186/1471-2458-11-319 PMC3112132 21575207

[27] Allen SE, Grimshaw HM, Rowland AP (1986) Chemical analysis, in: Moore, P.D., Chapman, S.B. (Eds.), Methods in Plant Ecology. Oxford, London: Blackwell Scientific Publication.

[28] Altshul L, Covaci A, Hauser R (2004) The relationship between levels of PCSs and pesticides in human hair and blood: preliminary results. Environ Health Perspect 112: 1193–1199. 15289166 10.1289/ehp.6916 PMC1247481

[29] US EPA (United States Environmental Protection Agency) (1997) Exposure factors handbook. EPA/600/P-95/002F. Washington, DC.

[30] Ma WJ, Deng F, Xu YJ, Xu HF, Nie DP (2005) The study on dietary intake and nutritional status of residents in Guangdong, South China. J Prev Med 31: 1–5 (in Chinese)..

[31] Zhai FY, He YN, Ma GS, Li YP, Wang ZH, et al (2005) Study on the current status and trend of food consumption among Chinese population. Chin J Epidemiol 26(7): 485–488 (in Chinese).. 16334997

[32] US EPA (United States Environmental Protection Agency) (2007) Integrated Risk Information System-database. Philadelphia PA; Washington, DC.

[33] Rodríguez L, Ruiz E, Alonso-Azcárate J, Rincón J (2009) Heavy metal distribution and chemical speciation in tailings and soils around a Pb–Zn mine in Spain. J Environ Manage 90: 1106–1116. 18572301 10.1016/j.jenvman.2008.04.007

[34] Lee JS, Chon HT, Kim KW (2005) Human risk assessment of As, Cd, Cu and Zn in the abandoned metal mine site. Environ Geochem Health 27: 185–191. 16003586 10.1007/s10653-005-0131-6

[35] Nobuntou W, Parkpian P, Kim Oanh NT, Noomhorm A, Delaune RD, et al (2010) Lead distribution and its potential risk to the environment: Lesson learned from environmental monitoring of abandon mine. J Environ Sci Health A Tox/Hazard Subst Environ Eng 45: 1702–1714. 10.1080/10934529.2010.513232 20853202

[36] MHPRC (Ministry of Health of the People’s Republic of China) (2012) The limits of pollutants in foods (GB 2762–2012) (in Chinese). Beijing, China: MHPRC.

[37] Lee CG, Chon HT, Jung MC (2001) Heavy metal contamination in the vicinity of the Daduk Au-Ag-Pb-Zn mine in Korea. Appl Geochem 16: 1377–1386.

[38] Ji K, Kim J, Lee M, Park S, Kwon HJ, et al (2013) Assessment of exposure to heavy metals and health risks among residents near abandoned metal mines in Goseong, Korea. Environ Pollut 178: 322–328. 23603469 10.1016/j.envpol.2013.03.031

[39] Moiseenko TI, Kudryavtseva LP (2001) Trace metal accumulation and fish pathologies in areas affected by mining and metallurgical enterprises in the Kola Region, Russia. Environ Pollut 114: 285–297. 11504351 10.1016/s0269-7491(00)00197-4

[40] Chen A, Lin C, Lu W, Wu Y, Ma Y, et al (2007) Well water contaminated by acidic mine water from the Dabaoshan Mine, South China: Chemistry and toxicity. Chemosphere 70: 248–255. 17910972 10.1016/j.chemosphere.2007.06.041

[41] Adhikari S, Ghosh L, Ayyappan S (2006) Combined effects of water pH and alkalinity on the accumulation of lead, cadmium and chromium to *Labeo rohita* (Hamilton). Int J Env Sci Tech 3: 289–296.

[42] Borgmann U, Couillard Y, Grapentine LC (2007) Relative contribution of food and water to 27 metals and metalloids accumulated by caged *Hyalella azteca* in two rivers affected by metal mining. Environ Pollut 145: 753–765. 16839651 10.1016/j.envpol.2006.05.020

[43] Proctor PD (1984) Heavy metal additions to the environment near mines, mills, and smelters, Southeast Missouri. In: Nriagu, J.O. (Ed.), Environmental Impacts of Smelters. Wiley, New York; 89–115.

[44] Li QS, Chen Y, Fu HB, Cui ZH, Shi L, et al (2012) Health risk of heavy metals in food crops grown on reclaimed tidal flat soil in the Pearl River Estuary, China. J Hazard Mat 227–228: 148–154. 10.1016/j.jhazmat.2012.05.023 22657103

[45] Albering HJ, van Leusen SM, Moonen EJC, Hoogewerff JA, Kleinjans JCS (1999) Human health risk assessment: A case study involving heavy metal soil contamination after the flooding of the river Meuse during the winter of 1993–1994. Environ Health Perspect 107: 37–43. 10.1289/ehp.9910737 PMC1566294 9872715

[46] Golia EE, Dimirkou A, Mitsios IK (2008) Influence of some parameters on heavy metals accumulation by vegetables grown in agricultural soils of different soil orders. B Environ Contam Toxicol 81: 80–84. 10.1007/s00128-008-9416-7 18431523

[47] McLaughlin MJ, Whatmuff M, Warne M, Heemsbergen D, Barry G, et al (2006) A field investigation of solubility and food chain accumulation of biosolid-cadmium across diverse soil types. Environ Chem 3: 428–432.

[48] Rodrigues SM, Pereira ME, Duarte AC, Römkens PF (2012) Soil-plant-animal transfer models to improve soil protection guidelines: a case study from Portugal. Environ Int 39: 27–37. 22208740 10.1016/j.envint.2011.09.005

[49] Zheng N, Wang QC, Zhang XW, Zheng DM, Zhang ZS, et al (2007) Population health risk due to dietary intake of heavy metals in the industrial area of Huludao city, China. Sci Total Environ 383: 81–89. 17765948 10.1016/j.scitotenv.2007.07.044

[50] Rieuwert JS, Farago ME, Cikrt M, Bencko V (2000) Differences in lead bioavailability between a smelting and a mining area. Water Air Soil Pollut 122: 203–229.

[51] Alonso E, Cambra K, Martinez T (2001) Lead and cadmium exposure from contaminated soil among residents of a farm area near an industrial site. Arch Environ Health 56: 278–282. 11480506 10.1080/00039890109604454

[52] Paoliello M, De Cpitani E, Cunha F, Matsuno T, Carvalho M (2002) Exposure of children to lead and cadmium from a mining area of Brazil. Environ Res 88: 120–128. 11908937 10.1006/enrs.2001.4311

[53] Cui YJ, Zhu YG, Zhai RH, Chen DY, Huang ZH, et al (2004) Transfer of metals from soil to vegetables in an area near a smelter in Nanning, China. Environ Int 30: 785–791. 15120196 10.1016/j.envint.2004.01.003

[54] Lim HS, Lee JS, Chon HT, Sager M (2008) Heavy metal contamination and health risk assessment in the vicinity of the abandoned Songcheon Au-Ag mine in Korea. J Geochem Explor 96: 223–230.

[55] Sun HF, Li YH, Ji YF, Yang LS, Wang WY, et al (2010) Environmental contamination and health hazard of lead and cadmium around Chatian mercury mining deposit in western Hunan Province, China. Trans Nonferrous Met Soc China 20: 308–314.

[56] Wcisło E, Ioven D, Kucharski R, Szdzuj J (2002) Human health risk assessment case study: an abandoned metal smelter site in Poland. Chemosphere 47(5): 507–515. 11996126 10.1016/s0045-6535(01)00301-0

[57] Zhou JM, Dang Z, Cai MF, Liu CQ (2007) Soil heavy metal pollution around the Dabaoshan mine, Guangdong province, China. Pedosphere 17: 588–94.

[58] Zhuang P, Li ZA, McBride MB, Zou B (2013) Health risk assessment for consumption of fish originating from ponds near Dabaoshan mine, South China. Environ Sci Pollut Res 20(8): 5844–5854. 10.1007/s11356-013-1606-0 23508535

[59] Hough RL, Breward N, Young SD, Crout NM, Tye AM, et al (2004) Assessing potential risk of heavy metal exposure from consumption of home-produced vegetables by urban populations. Environ Health Perspect 112: 215–221. 14754576 10.1289/ehp.5589 PMC1241831

[60] Pereira R, Ribeiro R, Goncalves F (2004) Scalp hair analysis as a tool in assessing human exposure to heavy metals (S. Domingos mine, Portugal). Sci Total Environ 327: 81–92. 15172573 10.1016/j.scitotenv.2004.01.017

[61] Waalkes MP (2003) Cadmium carcinogenesis. Mutat Res 533: 107–120. 14643415 10.1016/j.mrfmmm.2003.07.011

[62] Abrahams PW (2002) Soils: their implications to human health. Sci Total Environ 291: 1–32. 12150429 10.1016/s0048-9697(01)01102-0

[63] Branan N (2008) Mining leaves nasty legacy in Zambia. Geotimes website. Available: http://www.geotimes.org/jan08/article.html?id=nn_zambia.html. Accessed 2008 Jan.

[64] UNEP/OCHA (2010) Lead Pollution and Poisoning Crisis, Environmental Emergency Response Mission, Zamfara State, Nigeria, United Nations Environment Program, September/October.

[65] Thomas R (1980) Arsenic pollution arising from mining activities in south-west England. Inorganic Pollution and Agriculture. London: HMSO, 142–158.

