# Supplementary material for: Multiple Exposure and Effects Assessment of Heavy Metals in the Population near Mining Area in South China
Source: PLoS One. 2014 Apr 11;9(4):e94484. doi: 10.1371/journal.pone.0094484 (PMC3984172; doi:10.1371/journal.pone.0094484)
Supplement: Table S1 — Number of soil, rice, vegetable, soybean, water, sediment, fish and chicken samples collected from different sampling sites in the present study. (DOCX) [file pone.0094484.s002.docx]

**Table S1**

Number of soil, rice, vegetable, soybean, water, sediment, fish and chicken samples collected from different sampling sites in the present study.

| Subjects | SX | DS | FD | LQ | SB | XJ |
| --- | --- | --- | --- | --- | --- | --- |
| Soil | 18 | 24 | 24 | 18 | 20 | 18 |
| Rice | 8 | 10 | 10 | 8 | 10 | 8 |
| Vegetables | 48 | 60 | 58 | 46 | 60 | 48 |
| Soybean | –^a^ | 15 | 15 | – | – | – |
| Well water | – | 8 | 8 | – | 8 | – |
| Sediment | – | – | 12 | – | – | – |
| Fish | – | – | 152 | – | – | – |
| Chicken | – | – | 24(24)^b^ | – | – | – |
| Hair |  | 24 | 24(16)^c^ |  |  |  |

a No sample was collected.

b the number in parentheses was control samples.

c the number in parentheses was those from non-exposed population.
