# Supplementary material for: Multiple Exposure and Effects Assessment of Heavy Metals in the Population near Mining Area in South China
Source: PLoS One. 2014 Apr 11;9(4):e94484. doi: 10.1371/journal.pone.0094484 (PMC3984172; doi:10.1371/journal.pone.0094484)
Supplement: Table S2 — The edible part and water content of vegetables. (DOCX) [file pone.0094484.s003.docx]

**Table S2** The edible part and water content of vegetables

| Plant species | The edible tissue of the vegetable | Water content (%) |
| --- | --- | --- |
| *Brassica juncea L.* (mustard) | Leaves | 92.9 |
| *Brassica oleracea L.* (kale) | Leaves | 94.5 |
| *Brassica chinensis L.* (pak choi) | Leaves | 94.2 |
| *Brassica pekinensis (Lour.) Rupr.* (Chinese cabbage) | Leaves | 94.2 |
| *Brassica napus L.* (cole) | Leaves | 94.5 |
| *Brassica oleracea var. capitata* (cabbage) | Leaves | 93.5 |
| *Lactuca sativa var. romana Gars* (romaine lettuce) | Leaves | 95.2 |
| *Lactuca sativa var. longifolia Lam* (lettuce) | Leaves | 95.1 |
| *Spinacia oleracea L.* (spinach) | Leaves | 91.8 |
| *Apium graveolens L.* (celery) | Leaves + stalk | 94.1 |
| *Ipomoea aquatica L.* (water spinach) | Leaves | 92.5 |
| *Phaseolus vulgaris L.* (green bean) | Fruit | 90.2 |
| *Pisum sativum L.* (Garden pea) | Fruit | 88.9 |
| *Dolichos lablab L.* (dolichos bean ) | Fruit | 89.5 |
| *Lycopersicon esculentum L.* (tomato) | Fruit | 94.5 |
| *Capsicum annuum L.* (capsicum) | Fruit | 90.5 |
| *Solanum melongena L.* (aubergine) | Fruit | 93.1 |
| *Momordica charantia L.* (bitter melon) | Fruit | 92.8 |
| *Nelumbo nucifera Gaertn.* (lotus) | Root | 88.5 |
| *Raphanus sativus L.* (radish) | Root | 90.5 |
| *Daucus carota L.* (carrot) | Root | 88.6 |
| *Colocasia esculenta L.* (taro) | Root | 69 |
| *Ipomoea batatas L.* (sweet potato) | Tuber | 69 |
| *Dioscorea alata L.* (yam) | Tuber | 68 |
