# Supplementary material for: Multiple Exposure and Effects Assessment of Heavy Metals in the Population near Mining Area in South China
Source: PLoS One. 2014 Apr 11;9(4):e94484. doi: 10.1371/journal.pone.0094484 (PMC3984172; doi:10.1371/journal.pone.0094484)
Supplement: Table S3 — The number of fish, total length, fresh weight (fw) and habitat of fish samples. (DOCX) [file pone.0094484.s004.docx]

**Table S3**

The number of fish, total length, fresh weight (fw) and habitat of fish samples

|  | Species | N | Total length (cm) | Weight (g) | Age (y) | Habitat |
| --- | --- | --- | --- | --- | --- | --- |
| *Site1* | *Hypophthalmichthys molitrix* | 10 | 43.2 ± 0.95 | 935 ± 0.06 | 2 | Middle upper |
|  | *Ctenopharyngodon idellus* | 11 | 39.2 ± 0.51 | 686 ± 0.04 | 2 | Middle lower |
|  | *Megalobrama amblycephala* | 10 | 28.1 ± 1.2 | 289 ± 26.9 | 2 | Middle lower |
| *Site 2* | *C. idellus* | 8 | 58.0 ± 2.16 | 2090 ± 31.7 | 3 | Middle lower |
|  | *Aristichthys nobilis* | 6 | 62.2 ± 2.21 | 2642 ± 43.8 | 3 | Middle upper |
|  | *Cyprinus carpio* | 6 | 40.9 ± 2.53 | 813 ± 19.5 | 3 | Bottom |
| *Site3* | *H. molitrix* | 5 | 30.5 ± 3.5 | 355 ± 10.6 | 1 | Middle upper |
|  | *A. nobilis* | 7 | 45.3 ± 3.3 | 836 ± 18.5 | 1 | Middle upper |
|  | *Carassius* *auratus auratus* | 10 | 21.4 ± 2.6 | 161 ± 5.5 | 1 | Bottom |

Sampled fish included silver carp (*Hypophthalmichthys molitrix*), grass carp (*Ctenopharyngodon idellus*), wuchang carp (*Megalobrama amblycephala*), bighead carp (*Aristichthys nobilis*), and crucian carp (*Carassius* *auratus auratus*) and common carp (*Cyprinus carpio)*.
