# Supplementary material for: Multiple Exposure and Effects Assessment of Heavy Metals in the Population near Mining Area in South China
Source: PLoS One. 2014 Apr 11;9(4):e94484. doi: 10.1371/journal.pone.0094484 (PMC3984172; doi:10.1371/journal.pone.0094484)
Supplement: Table S4 — Heavy metal concentrations (mg/kg, mean ± SD) in the metal-enriched rice grain and chickenfeed for both groups of chicken. (DOCX) [file pone.0094484.s005.docx]

**Table S4**

Heavy metal concentrations (mg/kg, mean ± SD) in the metal-enriched rice grain and chickenfeed for both groups of chicken.

|  | Pb | Cd | Zn | Cu |
| --- | --- | --- | --- | --- |
| Rice grain | 1.27 ± 0.41 | 0.24 ± 0.03 | 37.8 ± 6.01 | 5.97 ± 1.15 |
| Chickenfeed | 0.29 ± 0.04 | 0.013 ± 0.001 | 121 ± 10.1 | 17.8 ± 3.64 |

Chicken feeding experiment:

The chicken feeding experiment lasted for 6 months. Forty-eight chicks (100–150 g) were divided into two groups. One group (the experimental group) was fed with metal-enriched rice grain grown in contaminated soil at Fandong village, and another group was only fed with chickenfeed served as control.
