# Supplementary material for: Multiple Exposure and Effects Assessment of Heavy Metals in the Population near Mining Area in South China
Source: PLoS One. 2014 Apr 11;9(4):e94484. doi: 10.1371/journal.pone.0094484 (PMC3984172; doi:10.1371/journal.pone.0094484)
Supplement: Table S5 — Average considered daily intakes of adults and children in the present study. (DOCX) [file pone.0094484.s006.docx]

**Table S5**

Average considered daily intakes of adults and children in the present study.

| Consumption ^a^  （g/person/d） | Rice | Leafy-vege. | Non-leafy vege. | Bean | Chicken | Fish | Water (L/d) | Soil |
| --- | --- | --- | --- | --- | --- | --- | --- | --- |
| Adult | 372 | 174 | 100 | 17 | 18 | 33.5 | 1.88 | 0.05 |
| Children | 198 | 68 | 40 | 16 | 12 | 17 | 1.50 | 0.15 |

^a^ A questionnaire-based survey was conducted in the studied villages to determine key risk factors such as dietary behaviors, daily activities and lifestyle of local people. We invited 50 local residents in each village to participate in the survey. Moreover, the consumption was estimated based on the dietary intake survey by Ma et al. (2005) and Zhai et al. (2005).
