# Supplementary material for: Multiple Exposure and Effects Assessment of Heavy Metals in the Population near Mining Area in South China
Source: PLoS One. 2014 Apr 11;9(4):e94484. doi: 10.1371/journal.pone.0094484 (PMC3984172; doi:10.1371/journal.pone.0094484)
Supplement: Table S6 — Standard reference materials for respective sample under investigation. (DOCX) [file pone.0094484.s007.docx]

**Table S6**

Standard reference materials for respective sample under investigation.

|  | Soil/sediment | Rice | Vegetable | Soybean | Fish | Chicken |
| --- | --- | --- | --- | --- | --- | --- |
| SRM | Contaminated soil  GBW08303 | Rice powder GBW08502 | Tea leaves GBW07605 | Tea leaves  GBW 10016 | *Pseudosciaena crocea* GBW 08573 | Pig muscle  GBW 08552 |
| Recovery rates | 98-101% | 95-105% | 96-105% | 94-103% | 93-102% | 96-102% |
