# Supplementary material for: Multiple Exposure and Effects Assessment of Heavy Metals in the Population near Mining Area in South China
Source: PLoS One. 2014 Apr 11;9(4):e94484. doi: 10.1371/journal.pone.0094484 (PMC3984172; doi:10.1371/journal.pone.0094484)
Supplement: Table S7 — The pH and organic matter (OM) of paddy and garden soils from Dabaoshan mine. (DOCX) [file pone.0094484.s008.docx]

**Table S7**

The pH and organic matter (OM) of paddy and garden soils from Dabaoshan mine

| Site | Type | pH (H_2_O) | OM (%) |
| --- | --- | --- | --- |
| SX | paddy soil | 4.57 | 2.73 |
|  | garden soil | 5.59 | 3.29 |
| DS | paddy soil | 5.14 | 3.16 |
|  | garden soil | 5.4 | 3.43 |
| FD | paddy soil | 5.24 | 3.45 |
|  | garden soil | 5.93 | 3.34 |
| LQ | paddy soil | 4.76 | 3.12 |
|  | garden soil | 6.89 | 3.83 |
| SB | paddy soil | 4.76 | 4.27 |
|  | garden soil | 5.88 | 2.38 |
| XJ | paddy soil | 4.54 | 2.93 |
|  | garden soil | 6.45 | 3.63 |

The six sampled villages are Shaxi (SX), Dongshan (DS), Fandong (FD), Liangqiao (LQ), Shangba (SB) and Xinjiang (XJ).
