# Supplementary material for: Multiple Exposure and Effects Assessment of Heavy Metals in the Population near Mining Area in South China
Source: PLoS One. 2014 Apr 11;9(4):e94484. doi: 10.1371/journal.pone.0094484 (PMC3984172; doi:10.1371/journal.pone.0094484)
Supplement: Supporting Information S1 — Description of study area. (DOCX) [file pone.0094484.s009.docx]

Supporting information for

**Multiple exposure and effects assessment of heavy metals in the population near mining area in South China**

Ping Zhuang, Huanping Lu, Zhian Li*, Bi Zou, Murray B. McBride

*Corresponding author. *E-mail address*: [lizan@scbg.ac.cn](mailto:lizan@scbg.ac.cn)

This file includes:

Description of study area

Table S1 to S7

Figure S1

**Study area**

Six sampling sites near the mine were selected as the study areas. Fandong (FD) village (24°33′78″ N; 113°43′94″ E), located on the mountaintop mine area (altitude at 500 m), was seriously affected by mining activities and atmospheric deposition. There was a mine tailing dam 0.5 km away from the village. Dongshan (DS, 24°35′72″ N; 113°39′94″ E) and Shaxi (SX, 24°35′79″ N; 113°42′34″ E) villages are located to the north of Dabaoshan mine, approximately 6 and 10 km away from the mine area, respectively. This area was contaminated by wastewater (Chuandu River) from the mining operation. Liangqiao (LQ, 24°35′79″ N; 113°42′34″ E), Shangba (SB, 24°27′83″ N; 113°48′16″ E) and Xinjiang (XJ, 24°26′81″ N; 113°49′14″ E) villages are located south of the Dabaoshan mine, along the Hengshi River, approximately 3, 12, and 14 km downslope away from the mine, respectively. The agricultural soil in this region was repeatedly irrigated with polluted water from the Hengshi and Chuandu Rivers.
